# Co-localization of influenza A virus and voltage-dependent calcium channels provides new perspectives on the internalization process in pigs

**DOI:** 10.1038/s44298-023-00009-x

**Published:** 2023-12-07

**Authors:** Charlotte Kristensen, Henrik E. Jensen, Ramona Trebbien, Pia Ryt-Hansen, Lars E. Larsen

**Affiliations:** 1 Department of Veterinary and Animal Sciences, University of Copenhagen, Frederiksberg C, Denmark; 2 Department of Virus and Microbiological Special Diagnostics, Statens Serum Institut, Copenhagen S, Denmark

**Keywords:** Influenza virus, Virus-host interactions, Viral pathogenesis

## Abstract

Influenza A virus (IAV) is an RNA virus that causes respiratory disease in a wide range of mammals including humans and pigs. Ca_v_1.2 is a specific voltage-dependent calcium channel (VDCC) important for the internalization of IAV and VDCC inhibitors can decrease IAV disease severity in mice. In this paper, the distribution pattern of a range of VDCCs by immunohistochemistry and Ca_v_1.2 by *in situ* hybridization in the porcine respiratory tract is documented for the first time. Furthermore, we showed co-localization of VDCC-positive and IAV-positive cells in experimentally infected pigs. These findings provide new perspectives on the IAV internalization process and pave the way for further research investigating the effect of VDDC inhibitors on the IAV infection dynamics in pigs, which could have relevance to humans too.

## Introduction

Influenza A virus (IAV) is a negative sense RNA virus that causes respiratory disease in humans and several animal species including pigs. The main IAV receptors are sialic acids (SA) that are linked to glycoproteins in either an alpha 2,3- (α2,3) or alpha 2,6-linkage (α2,6). IAVs isolated from humans and pigs prefer binding to SA-α2,6 while IAVs isolated from birds prefer binding to SA-α2,3^1–3^. Most IAVs seem to be species-specific and although humans and pigs have similar receptor distribution in their respiratory tract^4,5^, it is only some IAV strains that can be transmitted between pigs and humans^6^. This indicates that other factors are important for effective replication in a new host species. Such factors could be physical factors such as differences in temperatures, pH values, and host proteases in the respiratory tract^7–9^. Sporadic interspecies transmission of swine IAVs to humans most often results in dead-end transmissions, however, the most recent IAV pandemic (H1N1pdm09) occurred due to reassortment events in pigs^10,11^. The H1N1pdm09 became established in both pigs and humans and it has since then increased the diversity of swine IAVs dramatically, which has complicated the surveillance of IAVs^6,12,13^. Hence, a complete understanding of the swine and human IAV pathogenesis, to identify possible host factors that allow interspecies transmission, is needed to identify potential zoonotic swine IAVs.

Calcium channels have recently been shown to be important for the internalization of IAV and other human respiratory viruses, such as respiratory syncytial virus, measles, and rhinovirus^14^. Additionally, the presence of extracellular calcium is important for the internalization and replication of IAV, which has been demonstrated by adding ethylene glycol tetraacetic acid (EGTA, Ca^2+^ chelator)^15,16^ and by using specific voltage-dependent calcium channel (VDCC) blockers^17,18^. VDCCs consist of three families Ca_v_1 (L-type), Ca_v_2 (P/Q, N, and R-types), and Ca_v_3 (T-type). Common to all families of VDCCs is that stimulation by a voltage signal results in an extracellular calcium influx and they consist of an α1 subunit, which is the pore-forming unit of the VDCCs^19^. One of the L-type VDCC α1 subunits is Ca_v_1.2 and it contains four N-glycosylations sites, which can attach terminal SA^20^. Furthermore, it has been shown that hemagglutinin, the surface protein important for the attachment of IAV to cells, binds to Ca_v_1.2. Interestingly, this binding was significantly reduced by a sialidase pre-treatment, but not by adding EGTA, indicating that the sialic acids of Ca_v_1.2 are important for viral attachment^18^. Moreover, Fujioka et al.^18^ showed that a knockdown of Ca_v_1.2 significantly reduced IAV internalization. Overall, the previous studies strongly suggest that Ca_v_1.2 is an important factor in the internalization of IAV. Additionally, treatment of mice with Diltiazem, a specific Ca_v_1.2 inhibitor, significantly decreased the disease severity of IAV^18,21^. FDA has approved Diltiazem for the treatment of hypertension and coronary artery spasm^22^ and, therefore, a phase II clinical trial on Diltiazem as a treatment for severe IAV was already initiated in 2017^23^, however, the results of these trials are still pending.

Ca_v_1.2 has been detected by in situ methods in the lower respiratory tract in fetal lungs of humans and mice^24^, in adult cardiomyocytes of mice^25^ and rats^26^, and in adult murine brain tissues^21^. Furthermore, the Ca_v_1 and Ca_v_2 families of VDCCs have been recognized by Ca_v_Pan α1-antibodies in the adult murine lung by immunohistochemistry (IHC)^14^. In the present report, we document for the first time, the presence of Ca_v_1 and Ca_v_2 families by Ca_v_Pan α1-antibodies and specifically Ca_v_1.2 by in situ hybridization in the respiratory tract of pigs. These findings provide new perspectives on the swine IAV internalization process and open up for further investigation on the effect of VDCC-blockers as a treatment for IAV in pigs, which potentially could be translatable to humans.

## Methods

### Ethics declaration

The porcine and murine tissues originated from two experimental studies^27,28^ approved by the Danish Animal Experimentation Council (protocol no. 2020-15-0201-00502 and no. 2012-15-2934-00256, respectively).

### VDCC and IAV IHC

Immunohistochemically, Ca_v_Pan α1-antibodies stain the α1 genes from the Ca_v_1 and Ca_v_2 families and are therefore considered a pan-reactive VDCC reagent. The amino acid sequence used to produce the Ca_v_Pan α1-antibody corresponds to amino acids 1506 to 1524 of the rat Ca_v_1.2 and was compared to the corresponding amino acid sequences of Ca_v_1.2 from pig, mouse, human, guinea pig, ferret, chicken, and duck origin.

Heart, nose, trachea, and lung tissues from six to seven-week-old pigs (*N* = 3, one control pig and two IAV-inoculated pigs) were used for the examination of VDCC immunohistochemically, together with brain tissue from one adult mouse, which was used as a positive control. The formalin-fixed tissues were embedded in paraffin wax and sliced into 2–3 µm sections. The sections were deparaffinized and after each of the following steps, the sections were washed two times for 5 min in tris-buffered saline (TBS). The sections were blocked by endogen peroxidase 0.6% (CAS: 7722-84-1, VWR International, Søborg, Denmark), pre-treated with 0.1% trypsin, and blocked with ultra v-block (TL-125-HLJ, Thermo Scientific, Waltham, Massachusetts, USA). Ca_v_Pan α1-antibody (ACC-004, Almone Labs, Jerusalem, Israel) diluted 1:2000 was added to the sections overnight at 4 °C. The method of detection was Ultravision ONE HRP polymer reagent (TL-125-HLJ, Thermo Scientific) which was added to the sections for 30 min. A pre-adsorption control was performed as described above, but by mixing the Ca_v_Pan α1 blocking peptide (BLP-CC004, Almone Labs) and the Ca_v_Pan α1-antibody. An isotype control (IgG) (X0903, Agilent Technologies, Santa Clara, California, USA), diluted to the same content of protein as the primary reagent in 1% bovine serum albumin (BSA)/TBS, instead of Ca_v_Pan α1-antibody was also performed. The staining was developed by adding 3,3′-Diaminobenzidine (DAB) for 6 min, and sections were counterstained by Mayer’s hematoxylin (AMPQ00254.5000, VWR International, Radnor, Pennsylvania, USA) and mounted with glycerol-gelatine.

At least two representative images of each of the tissues and lung compartments (bronchi, bronchioles, and alveoli) were acquired. Lamina epithelialis in the nose, trachea, bronchi, bronchioles, and alveoli was manually selected as a region of interest (ROI) in ImageJ^29^. The percentage of pixels above the threshold in the selected area (% area) was calculated by using the plugin “Color Deconvolution2” and by setting a threshold of 126 for hematoxylin and 120 for DAB^30^. The % area of Ca_v_Pan α1 DAB was normalized to hematoxylin (% area DAB/% area hematoxylin) and the % area of isotype DAB normalized to hematoxylin was subtracted.

Parallel sections of two IAV-infected pigs, inoculated with an IAV circulating in pigs (swine-adapted) and an IAV circulating in humans (human-adapted), were stained for IAV in the nose, trachea, and lung tissues. The sections were deparaffinized and blocked for endogenous peroxidase as described above and hereafter pretreated with 0.018 g proteinase (P8038, Sigma-Aldrich, Missouri, USA) diluted in 100 ml TBS for 5 min. Additionally, the sections were blocked for 5 min by Ultra V Block (TA-125-UB, Epredia, Michigan, United States) and anti-IAV (nucleoprotein (NP)) antibodies (HYB 340-05, SSI-antibodies, Copenhagen S, Denmark) diluted 1:50000 in 1% BSA/TBS were added to the sections overnight at 4 °C. A Primary Antibody Enhancer (TL-125-PB, Epredia) was added to the sections for 20 min followed by a Large Volume HRP Polymer (TL-125-PH, Epredia) for 30 min. The staining was developed by adding AEC vector (SK-4200, Vector Laboratories, California, USA) for 10 min and counterstaining by Mayer’s hematoxylin (AMPQ00254.5000, VWR International). The sections were mounted with glycerol-gelatine.

### Cav.1.2 Reverse transcriptase-qPCR

The presence of Ca_v_1.2, in frozen lung tissue, obtained from three pigs that had tested negative for IAV, and in HEK 293 T cells was investigated. The HEK 293 T cells were used as a positive control for the reverse transcriptase-qPCR (RT-qPCR) assay since the Ca_v_1.2 channels previously have been documented in these cells^18^. Briefly, 200 µl of HEK 293 T cells with Eagle’s Minimum Essential Medium (MEM) (Gibco) were collected and treated as previously described for nasal swabs^31^. RLT-buffer (QIAGEN, Hilden, Germany) which contained 2-mercaptoethanol (Merck, Darmstadt, Germany) was added to 70 mg frozen lung tissues, lysed in TissueLyser LT (QIAGEN) at 30 Hz for 3 min and centrifuged at 9651 × *g* for 3 min. The RNA extraction was performed as described previously^31^. RT-qPCR was performed by using Qiagen one-step RT-PCR kit (QIAGEN, Hilden, Germany) with primers for Ca_v_1.2 developed by ref. ^18^. The PCR cycles were as follows: 50 °C for 40 min, 95 °C for 15 min, and 45 cycles of (94 °C 40 s, 60 °C 40 s, 72 °C 40 s) and 72 °C for 10 min. The amplified Ca_v_1.2 was detected by SYBR green.

### Cav1.2 Sanger sequencing

The RT-qPCR product was purified with a High Pure PCR product Purification kit (Roche Diagnostics GmbH, Mannheim, Germany) according to the manufacturer’s protocol. PCR products of one of the pig lung tissues together with primers (the same as used for the RT-qPCR) were sent for Sanger sequencing at LGC Biosearch Technologies (Berlin, Germany). The resulting sequences were imported into the program CLC Main Workbench version 22.0 (QIAGEN, Hilden, Germany) and trimmed manually. The forward and reverse reads were assembled and the consensus sequence was checked for identity to publically available sequences using the tool BLAST against NCBI Genbank. The sequence was uploaded to NCBI Genbank with the accession number OQ973297.

### In situ hybridization

The consensus sequences were used to create probes for the *in situ* hybridization to investigate the Ca_v_1.2 mRNA expression in porcine lung tissues (*N* = 1). The probe sequences correspond to nucleotides 3532–3625 (between exon 27-exon 28) on the *sus scrofa* calcium voltage-gated channel subunit alpha1 C/Ca_v_1.2 (CACNA1C) predicted coding nucleotide sequence Genbank ID XM_021092981.1 and were ordered from by Eurofins Genomics (Germany GmbH). The sequences were as follows; sense, 5′ GCT GGA CAA GAA CCA GCG GCA GTG ‘3 and antisense, 5′ CAC TGC CGC TGG TTC TTG TCC AGC 3′. The probes were labeled with CY3 fluorophore on both ends and HPLC purified. The in situ hybridization was performed using Shandon racks as earlier described with minor modifications^32^. The sections were pretreated with 10% Proteinase K diluted in TBS (124568, Sigma-Aldrich, Missouri, USA) for 10 min at 37 °C and flushed two times with Mili-Q water after deparaffinization. To investigate the amount of autofluorescent signal in the porcine tissues the method described above was performed but without adding the probes. The images of the porcine lung tissues were obtained with an LSM 900 microscope with an Airyscan2 detector attached and a 40x/1.2 objective. All images were acquired, processed, and analyzed with the ZEN software by using the same settings for the CY3 channel on all images. The images were analyzed in ImageJ in the same manner as described for Ca_v_Pan α1 IHC but by using the “split channels” function, selecting the surface of lamina epithelialis, and setting a threshold of 181 for DAPI and 6; 158 for the Ca_v_1.2 probes.

## Results

### VDDCs and Cav1.2 are expressed on the surface of the porcine respiratory epithelium

The HEK 293 T cells and the porcine lung tissues tested positive for Ca_v_1.2 when tested by RT-qPCR. The consensus sequence (OQ973297) of the porcine lung tissue corresponds to nucleotides 3532-3625 on *sus scrofa* calcium voltage-gated channel subunit alpha1 C/Ca_v_1.2 (CACNA1C) predicted coding nucleotide sequence (Genbank ID XM_021092981.1). The porcine consensus sequence had the highest sequence similarity with the human Ca_v_1.2 (97% identity) followed by the guinea pig and the ferret Ca_v_1.2 sequences (96 % and 95% identity, respectively, Supplementary file S1).

**Fig. 1:**
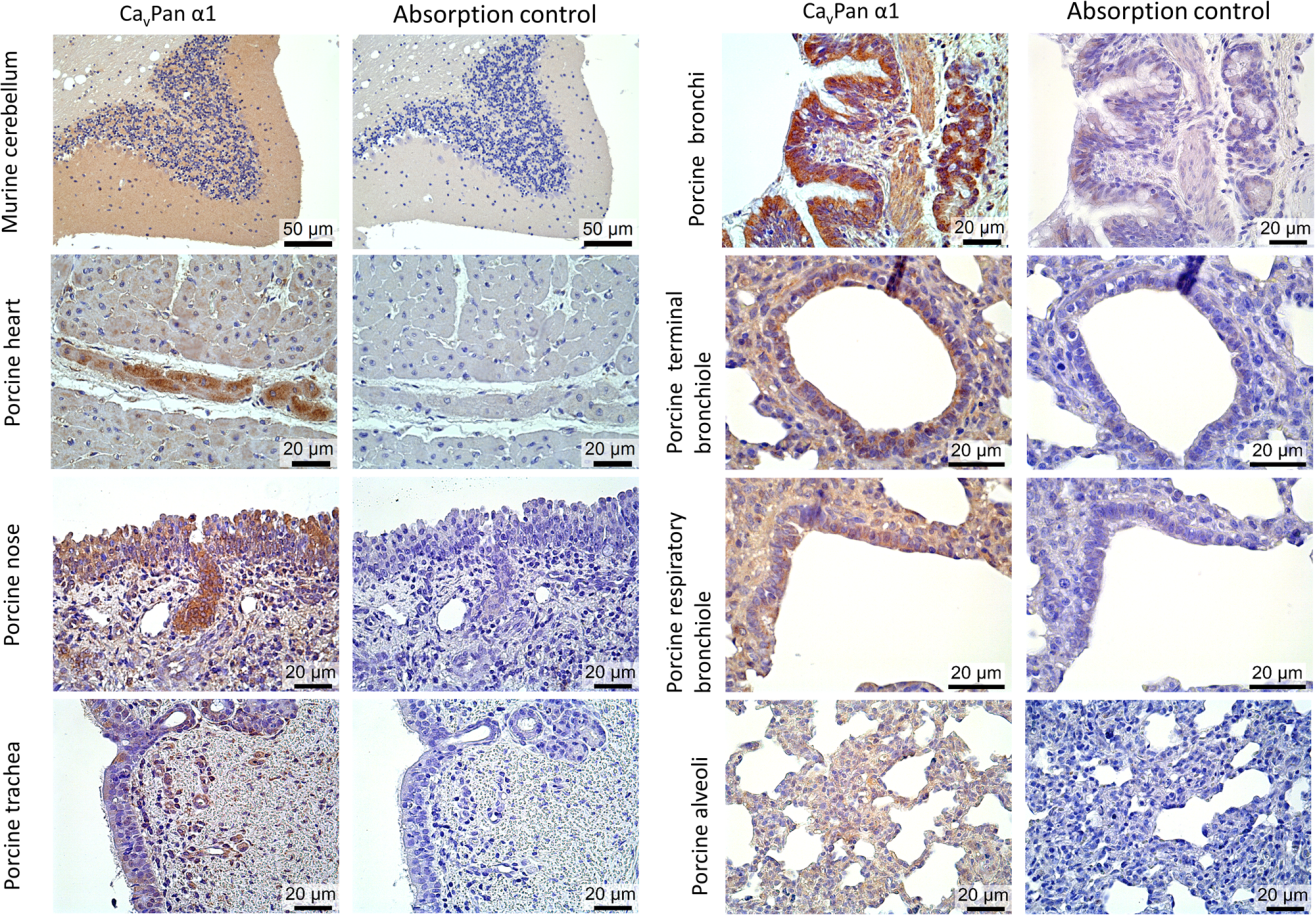
α1 subunits of Ca_v_1 and Ca_v_2 families (Ca_v_Pan α1) of the L-type voltage-dependent calcium channels are expressed in the murine brain, porcine heart, nasal, tracheal, and lung tissues. The presence of Ca_v_Pan α1 (brown staining) in murine brain, porcine heart, nasal, tracheal, and lung tissues was visualized immunohistochemically by CavPan α1-antibodies. A negative control was performed by a homologous absorption test on parallel sections of the murine brain together with the porcine heart and respiratory tissues.

**Fig. 2:**
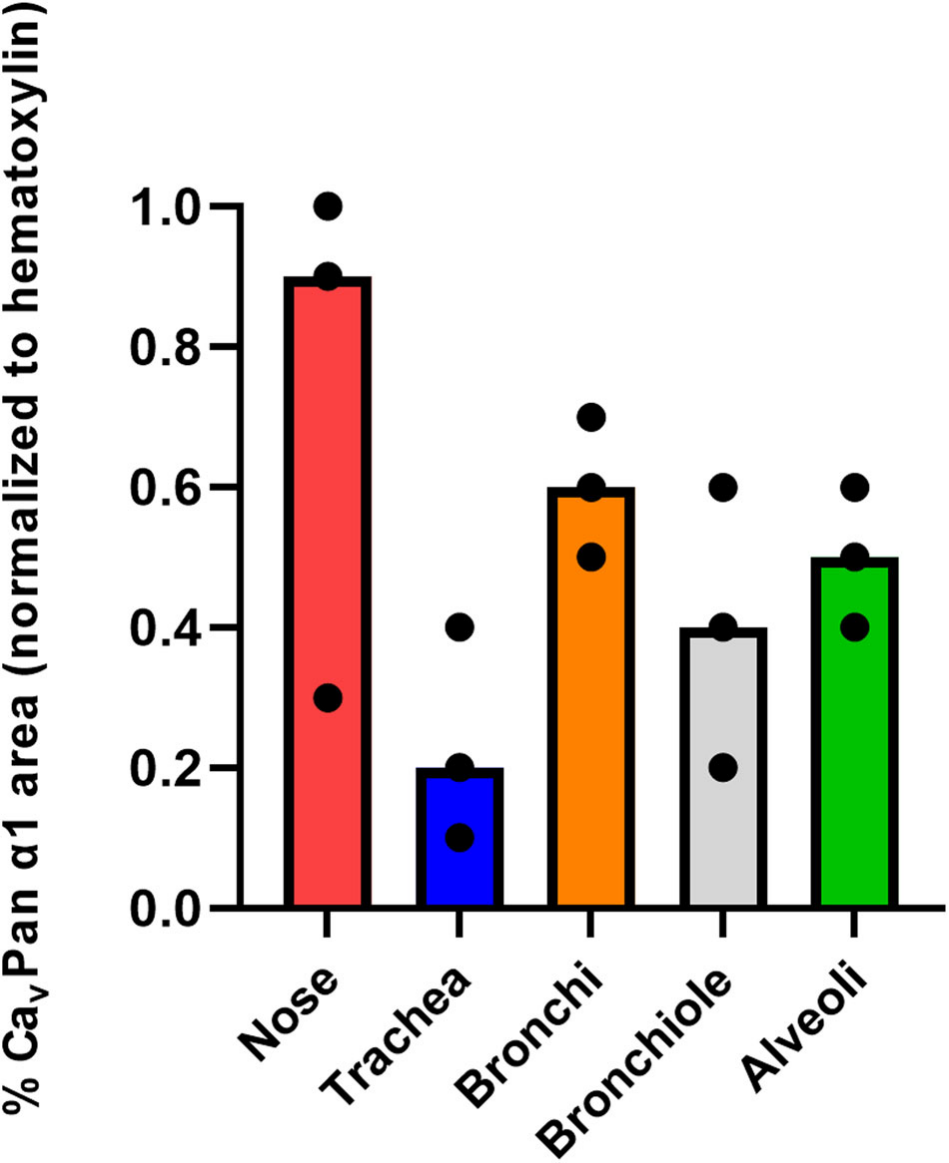
The lowest expression of α1 subunits of Ca_v_1 and Ca_v_2 families (Ca_v_Pan α1) is found in the porcine trachea. The median percentage of Ca_v_Pan α1 staining in the epithelium (% area (normalized to hematoxylin)) in the porcine respiratory tissues and different lung compartments.

All of the selected species showed 100% homology in the Ca_v_1.2 amino acid sequence targeted by the Ca_v_Pan α1 antibody (Supplementary file 2). The immunohistochemical staining for Ca_v_Pan α1 and the negative absorption control is shown in Fig. 1. The absorption control showed no staining in the murine brain and porcine heart, nasal and tracheal tissues, and only weak irregular staining was found in the bronchial epithelium. Furthermore, the application of the isotype IgG-antibody control in murine brain tissue and porcine heart, nose, trachea, and lung tissues is shown in Fig. S1. Although the isotype control was negative in the murine brain tissue, unspecific staining was observed in the porcine heart, nose, bronchi, and bronchioles, but it was significantly lower than the staining intensity of Ca_v_Pan α1. The Ca_v_Pan α1 staining of the murine brain showed strong staining in the molecular, granular, and Purkinje cell layers of the cerebellum and in neurons (Fig. 1). Furthermore, there was a moderate to strong positive staining of Ca_v_Pan α1 throughout the porcine heart tissue with the highest staining intensity of the Purkinje fibers. In the porcine respiratory tissues, the strongest staining was observed in the epithelial layer and the glands in the submucosal layer. Furthermore, there was also a weaker positive staining of the smooth muscle cells surrounding the bronchi and bronchioles. The % Ca_v_Pan α1 area in lamina epithelialis of the different porcine respiratory tissues and lung compartments is presented in Fig. 2. Briefly, the highest % Ca_v_Pan α1 area was observed in the nose and bronchi followed by the alveoli and the bronchioles, which also showed a higher variation of staining. The lowest expression of Ca_v_Pan α1 was observed in the trachea.

**Fig. 3:**
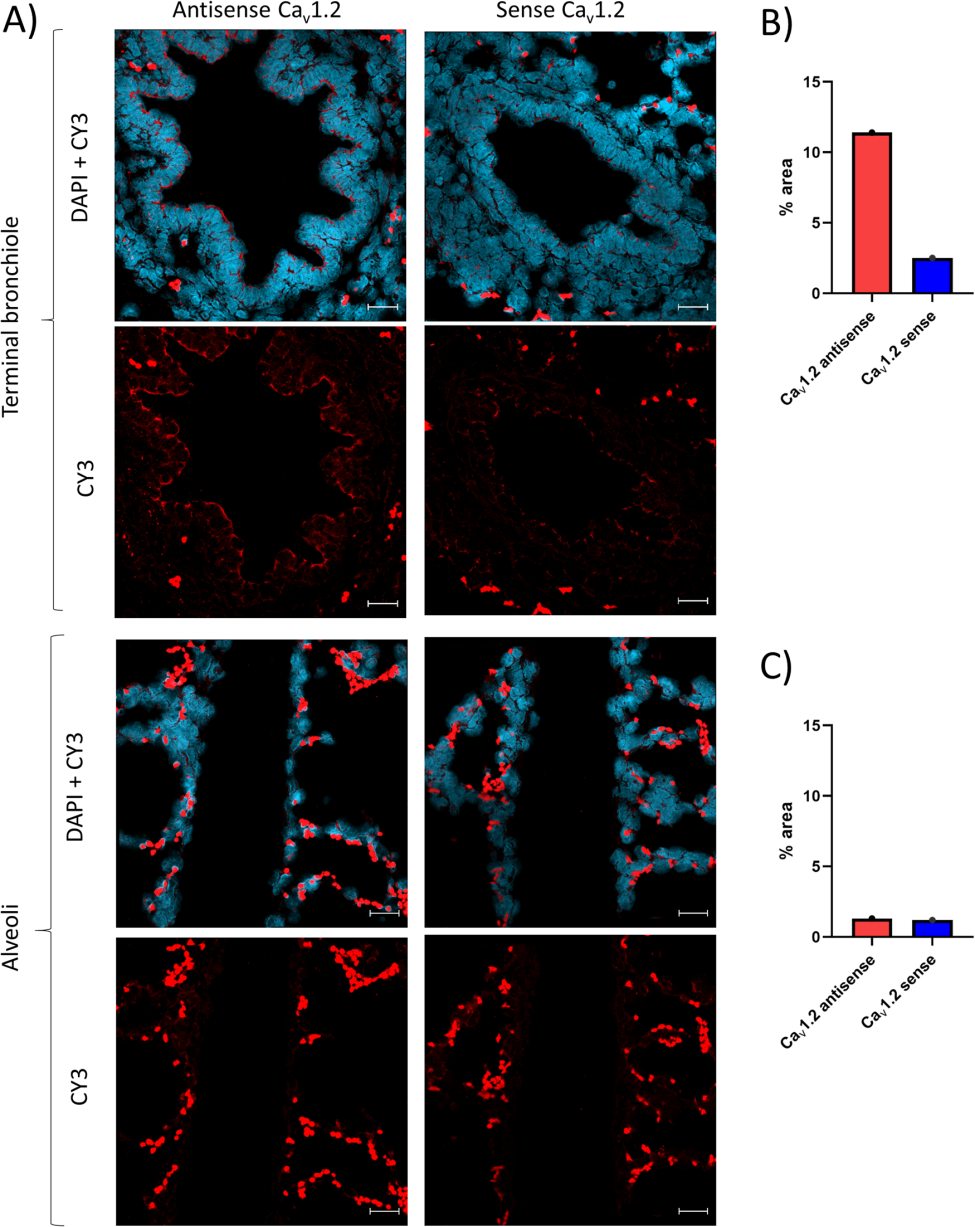
The Ca_v_1.2 L-type voltage-dependent calcium channels are expressed in the porcine terminal bronchioles. **A** The presence of Ca_v_1.2 (red staining) in porcine lung tissues was visualized by in situ hybridization and a CY3 fluorophore-labeled anti-sense Ca_v_1.2 probe. A negative control was performed using a sense probe. **B** The percentage of area with pixels above the threshold in the bronchiolar epithelium (% Ca_v_1.2 area) observed with the anti-sense and sense probes. **C** The percentage of area with pixels above threshold in the alveoli (% Ca_v_1.2 area) observed with the anti-sense and sense probes. Images were obtained by an LSM 900 microscope and an Airyscan2 detector. Blue: nuclear DAPI staining. White scale bar indicates 20 µm.

Ca_v_1.2 mRNA visualized by in situ hybridization is presented in Fig. 3. Positive staining on the surface of the terminal bronchioles epithelial cells was observed with the anti-sense probe, whereas the sense probe showed a weaker signal. The anti-sense and sense probes showed no to weak positive staining of the alveoli, and as no difference between the two probes was observed, this signal only was interpreted as a background signal. In general, an autofluorescent signal of the erythrocytes was observed but otherwise, no autofluorescent signal was found (Fig. S2).

### Cells expressing CavPan α1 co-localize with cells positive for IAV in experimentally infected pigs

**Fig. 4:**
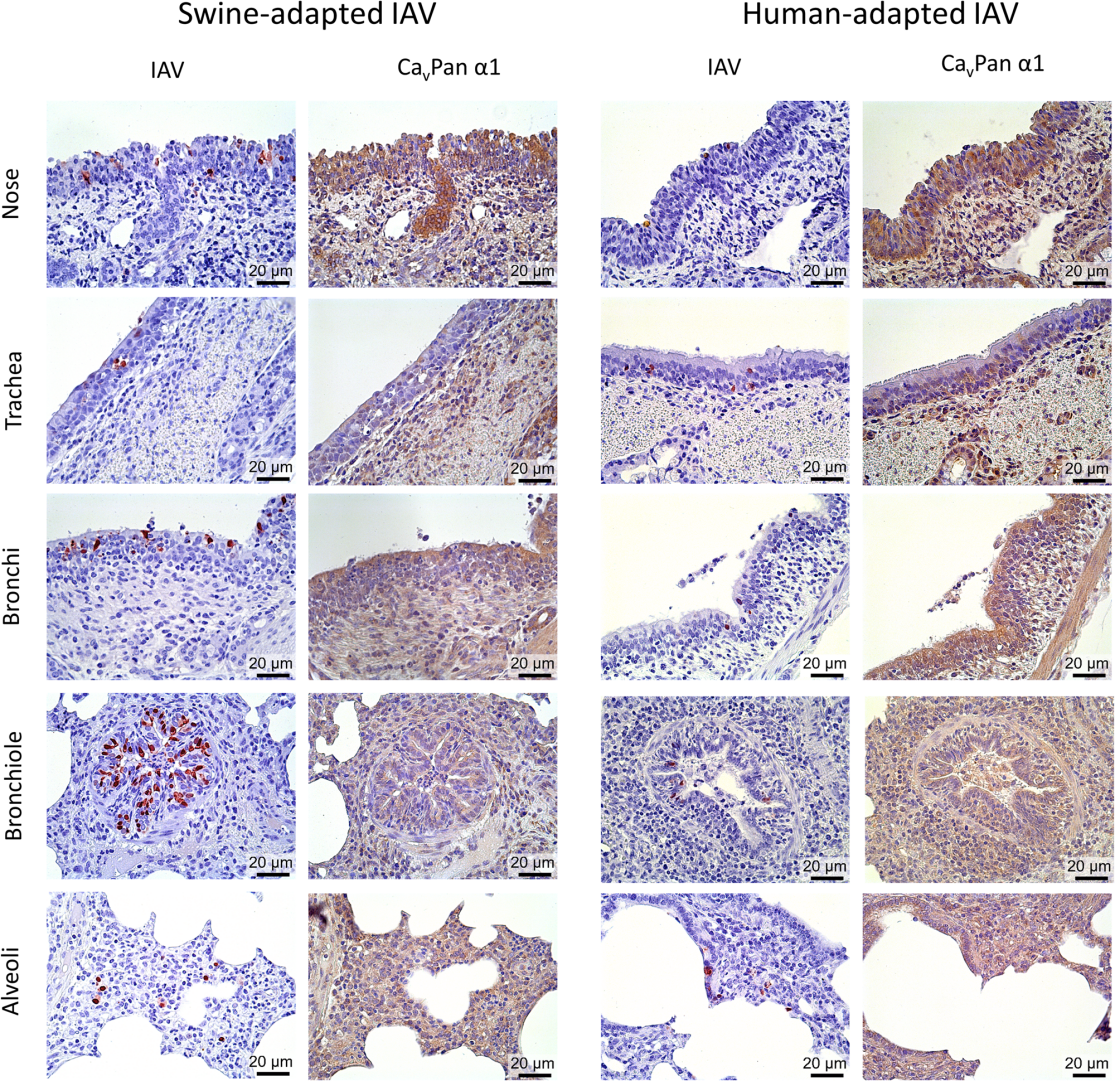
Co-localization of influenza A virus (IAV) and α1 subunits of Ca_v_1 and Ca_v_2 families (Ca_v_Pan α1). Parallel sections of IAV (red staining) and Ca_v_Pan α1 (brown staining) in the respiratory tract of a pigs experimentally infected with an IAV circulating in pigs (*N* = 1, swine-adapted) and an IAV circulating in humans (*N* = 1, human-adapted).

Parallel sections of immunohistochemical staining for IAV and Ca_v_Pan α1 from two IAV-experimentally infected pigs (one with swine-adapted IAV and one with human-adapted IAV) are presented in Fig. 4. Co-localization of IAV-positive and Ca_v_Pan α1-positive cells was present in the respiratory epithelium throughout the respiratory tract. Furthermore, the leukocytes present in the bronchial and bronchiolar exudate also stained positive for Ca_v_Pan α1 but were only rarely positive for IAV.

## Discussion

The most interesting finding was that α1 subunits of Cav1 and Cav2 families (CavPan α1) are expressed in the respiratory epithelium throughout the porcine respiratory tract and co-localize with IAV-positive cells in experimentally IAV-infected pigs. The expression of Ca_v_1.2 was observed in the porcine terminal bronchioles but not in the alveoli.

The anti-Ca_v_Pan α1 staining of the adult murine brain correlates to previous findings of non-fetal mice^25,33^, and the Ca_v_Pan α1 in the porcine heart is in agreement with findings in cardiomyocytes of adult mice^25^. Both staining methods showed staining of the porcine lungs that correlate to the findings in fetal human and fetal/adult murine lung tissue^18,24^. Anti-Ca_v_Pan α1 binds to both the Ca_v_1 (Ca_v_1.1–1.4) and Ca_v_2 families (Ca_v_2.1– 2.3) and showed staining of the smooth muscle cells surrounding the bronchi and bronchioles of the lungs, whereas the Ca_v_1.2 antisense-probe specifically binds to Ca_v_1.2 and showed no staining of the smooth muscle cells. This difference could be explained by Ca_v_2.2 showing a stronger staining intensity of the smooth muscle cells compared to Ca_v_1.2 in the fetal human lung, which only showed weak staining of the smooth muscle cells^24^.

The IAV tropism in pigs are epithelial cells throughout the respiratory tract, and occasionally in glands in the submucosal layer and alveolar macrophages^5,34–39^. The lowest expression of α1 subunits of Cav1 and Cav2 families (CavPan α1) was observed in the trachea, which correlates with a previous study that found a lower viral load in the trachea compared to porcine nose and lung tissues^40^. An explanation for a lower viral load in the trachea remains to be clarified since the pig IAV host receptor (SA-α2,6) is expressed in the trachea (5, 36, 38), however, one explanation could be a lower expression of α1 subunits of Cav1 and Cav2 families. Furthermore, a higher viral load is mainly found in the bronchiolar epithelium compared to the alveoli^5,35,36,38^, which correlates with the Ca_v_1.2 mRNA expression. Thus, Ca_v_1.2 specifically could be an important factor for IAV internalization in pigs. Further investigations of α1 subunits of Cav1 and Cav2 families and specifically Ca_v_1.2 distribution in the adult human lung tissues are needed to elucidate if pigs and humans have different distribution patterns in the respiratory tissues. Additionally, we observed a high within-tissue variation of the expression of α1 subunits of Cav1 and Cav2 families and if this is explained by variance in the IHC staining or due to other factors such as the presence of neutrophils expressing ca_v_2.3 and/or IAV-induced necrosis of the epithelial cells, remain unknown^41^.

The results of the present study strongly suggest that the pig is a suitable model to investigate VDCC inhibitors’ effect on the treatment of IAV in porcine cell lines. Additionally, optimized RT-qPCR or situ hybridization methods are needed to quantify the expression of Ca_v_1.2 throughout the porcine respiratory tract and to evaluate if Ca_v_1.2 is up- or down-regulated during an IAV infection. Squamous metaplasia is often induced after IAV-infections in humans and squamous metaplasia has been shown to decrease the expression of Ca_v_1.2 in the nasopharynx of humans, suggesting that Ca_v_1.2 is less expressed in humans with recent IAV infections^42,43^.

The co-localization of IAV and VDCCs might be translatable to humans since the pathogenesis of IAV infection in pigs resemble human, pigs show clinical signs similar to humans^44^, and humans also express Ca_v_1.2 in their fetal respiratory tract. Additionally, an L-type VDCC inhibitor prevents the internalization and spread of SARS-Cov-2, thus it could be speculated that Ca_v_1.2 is important for other respiratory viruses as well^45^.

## Supplementary information

Supplementary Information

## Data Availability

Images and ROIs used for the semi-quantification are uploaded to the Open Science Framework (https://osf.io/8fz6j/, 10.17605/OSF.IO/8FZ6J) otherwise data is presented within the paper or in the supplementary files.
