# Supplementary material for: Co-localization of influenza A virus and voltage-dependent calcium channels provides new perspectives on the internalization process in pigs
Source: Npj Viruses. 2023 Dec 7;1:8. doi: 10.1038/s44298-023-00009-x (PMC11721136; doi:10.1038/s44298-023-00009-x)
Supplement: Supplementary file 1 — Supplementary Information [file 44298_2023_9_MOESM1_ESM.pdf]

Supplementary File S1. Comparison between the nucleotide sequences of the porcine Ca_v1.2 obtained from this study and other relevant species. The porcine Ca_v1.2 nucleotide sequence was found by Sanger sequencing of the PCR product from the reverse transcriptase-qPCR of the porcine lung tissue.

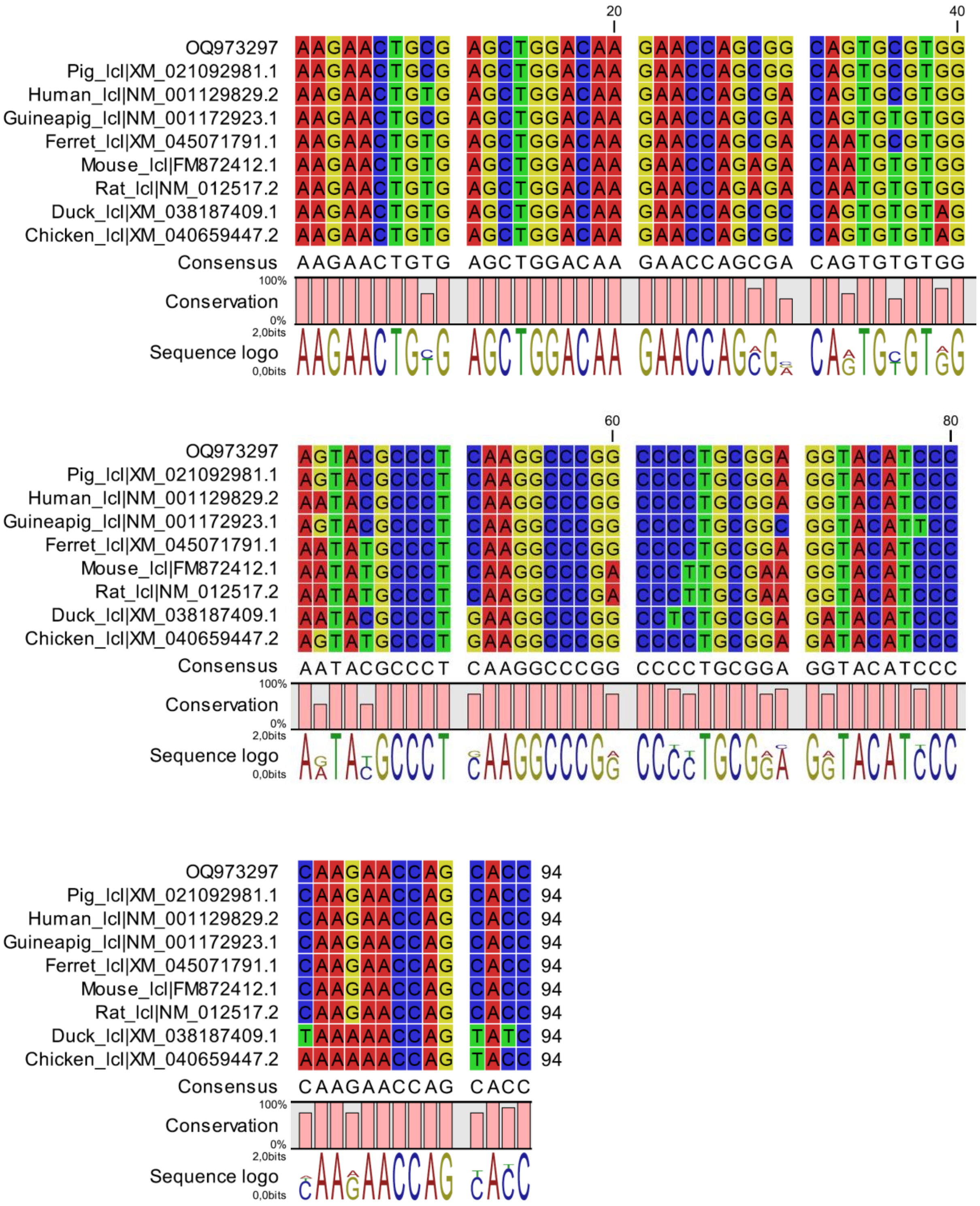

Supplementary File S2. Comparison of the Ca_v1.2 target epitope of the Ca_vPan α1-antibody between different species.

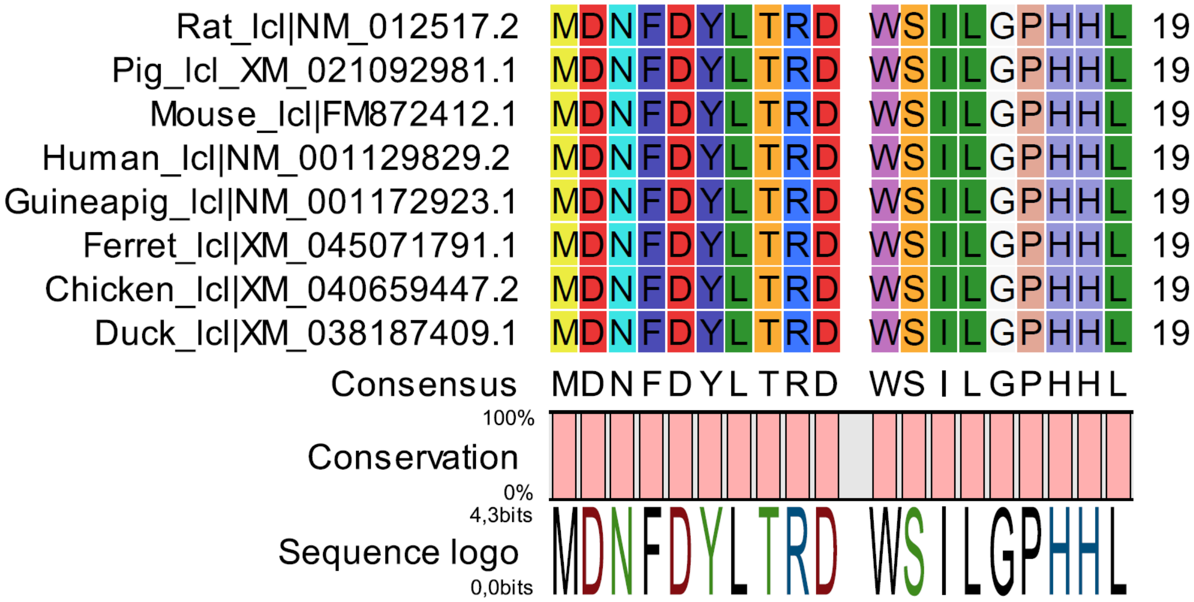

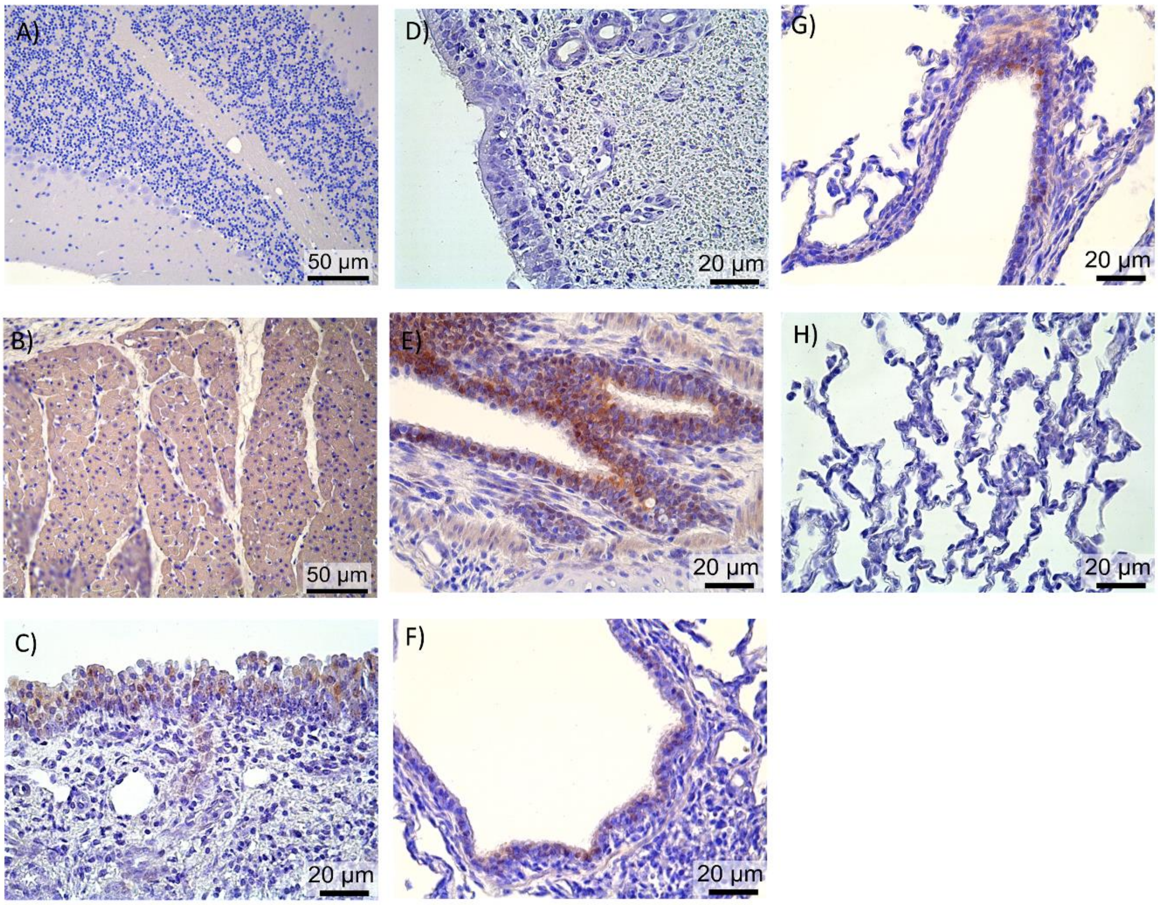

Figure S1. Weak to moderate unspecific binding in the porcine tissues. The presence of unspecific binding (brown staining) in the murine brain (A), porcine heart (B), porcine respiratory tissues (C-H) was investigated immunohistochemically by an isotype control. No unspecific staining was observed in the murine brain (A) or porcine trachea (D) and alveoli (H). Weak unspecific staining was observed in the epithelium of the porcine heart (B), nasal mucosa (C), bronchioles (F) and respiratory bronchioles (G). Moderate background staining of the bronchial epithelium and weak staining of the smooth muscle cells was observed in the porcine bronchi (E).

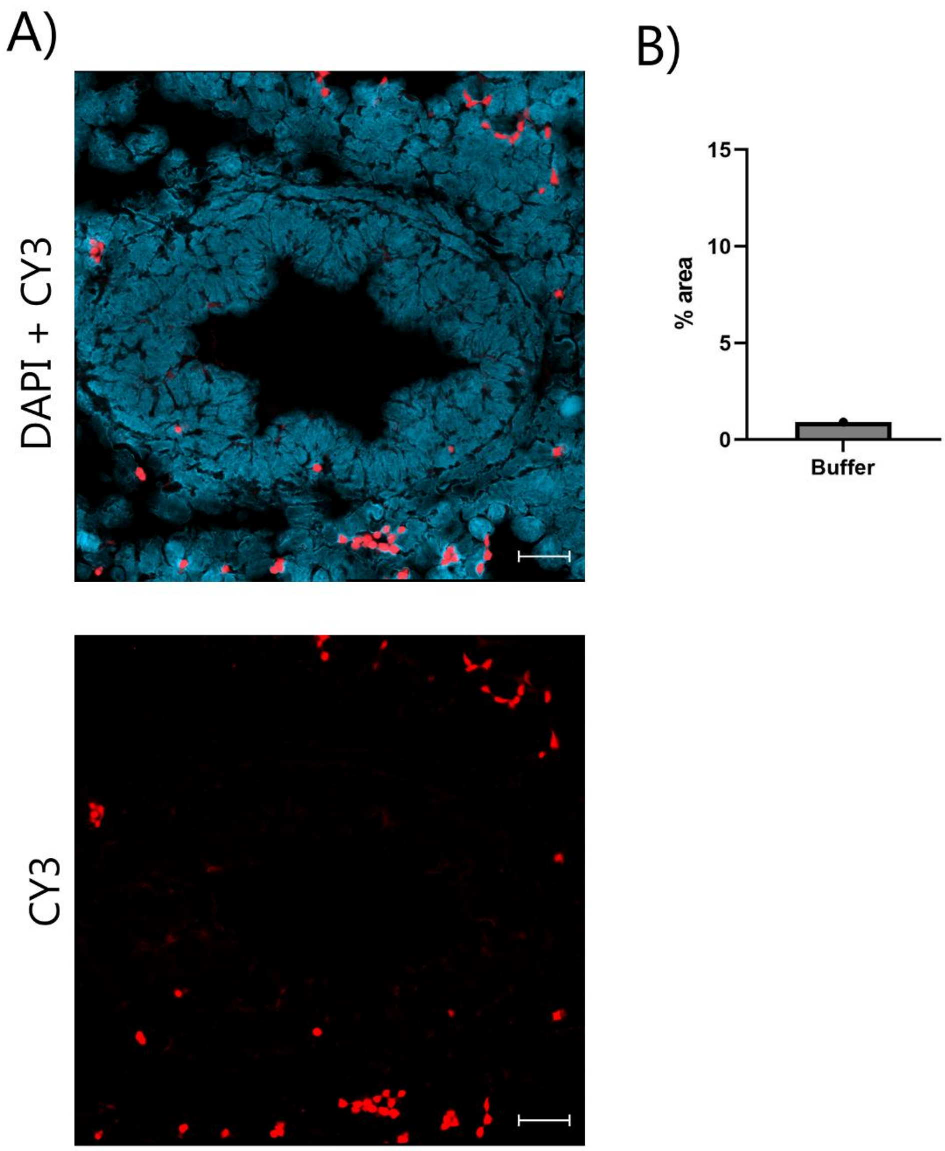

Figure S2. No autofluorescent signal was observed in the porcine lung tissue except for the autofluorescent erythrocytes. A) The presence of an autofluorescent signal of the porcine lung tissue was investigated by performing the in situ hybridization but without adding any probes only buffer. B) The percentage of area with pixels above threshold in the bronchiolar epithelium (% area). Pictures were obtained by an LSM 900 microscope and an Airyscan2 detector. Blue: nuclear DAPI staining. White scale bar indicates 20µm.
